# Resequencing of reindeer genomes provides clues to their docile habits

**DOI:** 10.1093/evlett/qrae006

**Published:** 2024-03-05

**Authors:** Baosheng Wu, Qingmiao Ren, Xiaoting Yan, Fei Zhao, Tao Qin, Peidong Xin, Xinxin Cui, Kun Wang, Rui Du, Knut H Røed, Steeve D Côté, Glenn Yannic, Zhipeng Li, Qiang Qiu

**Affiliations:** Shaanxi Key Laboratory of Qinling Ecological Intelligent Monitoring and Protection, School of Ecology and Environment, Northwestern Polytechnical University, Xi’an, China; Shaanxi Key Laboratory of Qinling Ecological Intelligent Monitoring and Protection, School of Ecology and Environment, Northwestern Polytechnical University, Xi’an, China; Shaanxi Key Laboratory of Qinling Ecological Intelligent Monitoring and Protection, School of Ecology and Environment, Northwestern Polytechnical University, Xi’an, China; College of Animal Science and Technology, Jilin Agricultural University, Changchun, China; Shaanxi Key Laboratory of Qinling Ecological Intelligent Monitoring and Protection, School of Ecology and Environment, Northwestern Polytechnical University, Xi’an, China; Shaanxi Key Laboratory of Qinling Ecological Intelligent Monitoring and Protection, School of Ecology and Environment, Northwestern Polytechnical University, Xi’an, China; Shaanxi Key Laboratory of Qinling Ecological Intelligent Monitoring and Protection, School of Ecology and Environment, Northwestern Polytechnical University, Xi’an, China; Shaanxi Key Laboratory of Qinling Ecological Intelligent Monitoring and Protection, School of Ecology and Environment, Northwestern Polytechnical University, Xi’an, China; Jilin Provincial Engineering Research Center for Efficient Breeding and Product Development of Sika Deer, Changchun, China; College of Chinese Medicine Materials, Jilin Agricultural University, Changchun, China; Department of Preclinical Sciences and Pathology, Norwegian University of Life Sciences, Ås, Norway; Département de Biologie, Caribou Ungava, Centre d’Études Nordiques, Université Laval, Québec, QC, Canada; Université Grenoble Alpes, Université Savoie Mont Blanc, CNRS, LECA, Grenoble, France; College of Animal Science and Technology, Jilin Agricultural University, Changchun, China; Shaanxi Key Laboratory of Qinling Ecological Intelligent Monitoring and Protection, School of Ecology and Environment, Northwestern Polytechnical University, Xi’an, China

**Keywords:** reindeer, domestication, docile, artificial selection

## Abstract

Reindeer have long been served as vital subsistence resources for inhabitants of Arctic and subarctic regions owing to their domestication. However, the evolutionary relationships and divergence times among different reindeer populations, genetic traits that distinguish domesticated reindeer, and factors that contribute to their relative docility compared with that of other Cervidae specie, remain unclear. In this study, we sequenced the genomes of 32 individuals from wild and domestic reindeer populations that inhabit Arctic and subarctic regions. We found that reindeer experienced 2 or more independent domestication events characterized by weak artificial selection pressure and limited significant differences in genomic parameters between wild and domestic populations. Alterations in conserved noncoding elements in the reindeer genomes, particularly those associated with nervous system development, may have contributed to their domestication by rendering the nervous system less responsive. Together, our results suggest that inherent species-specific traits, rather than intense artificial selection, may have played a significant role in the relatively docile behavior of reindeer and offer valuable insights into the domestication process of these animals.

## Introduction

Reindeer (*Rangifer tarandus*) inhabit a wide range of Arctic and subarctic areas, including the northern regions of North American (caribou) and Eurasia as well as large islands within cold forests and tundra (Holand et al., 2022; Williams & Heard, 1986). In North American, their range extends from Alaska through Yukon, the Northwest Territories, Nunavut, and down the Canadian Rocky Mountains (Flagstad & Røed, 2003). Similarly, Eurasian reindeer are found from Iceland in the west to Siberia in the east, and reach as far south as northeastern China (Flagstad & Røed, 2003; Krupnik, 2000; Pálsson et al., 1994; Wang et al., 2019). Since the Paleolithic era, reindeer hunting and herding have been crucial for the survival of human societies, and those practices continue to day (Pelletier et al., 2020; Salmi, 2022). Studies using mitochondrial DNA and paleontological data have shown the significant impact of the last glacial period (LGP), especially during the last glacial maximum (LGM), on the widespread distribution of reindeer in Arctic and subarctic regions (Anderson et al., 2017; Holand et al., 2022; Kvie et al., 2016; Weckworth et al., 2012). Archeological evidence suggests the beginning of reindeer domestication in the Mesolithic period, but other studies have indicated that it may have begun later, in the Late Iron Age (Helskog & Indrelid, 2011; Murashkin et al., 2016). The exact timeline of domestication varies among reindeer populations and remains uncertain because of the limited scope of the available archeological and genomic data (Pelletier et al., 2022; Salmi, 2022; van den Berg et al., 2023).

The domestication of reindeer has intrigued historians, archeologists, and ethnographers for over a century, leading to ongoing debates and research (Losey et al., 2021; Salmi, 2022; Storli, 1996). Studies of ancient and modern mitochondrial and microsatellite DNA from different reindeer populations indicate two main independent domestication events for Eurasian reindeer: one in Fennoscandia and one in Western Siberia (Pelletier et al., 2020; Røed et al., 2008, 2011). However, an analysis of 193 ancient mitochondrial DNA control regions from reindeer in northern Fennoscandia suggested that most reindeer in this area were introduced from elsewhere, casting doubt on the exact origins of domestication (Røed et al., 2018). Unlike Eurasian reindeer domestication, reindeer (caribou) in North America and Greenland were not bred domestically by indigenous peoples; rather, domestic reindeer was introduced from Siberia and Norway in the 19th and 20th centuries, respectively (Jepsen et al., 2002; Lantis, 1950; Mager, 2012). Investigating the genetic diversity in the genomes of both wild and domestic reindeer across different populations will help to unravel the complexities of the domestication process.

Besides the unique aspects of reindeer domestication, reindeer also exhibit a relatively docile nature within the Cervidae family (Feng et al., 2023; Wong et al., 2021). For example, forest musk deer (*Moschus berezovskii*) (Feng et al., 2023) and Eld’s deer (*Rucervus eldii*) (Wong et al., 2021) are known for their shy and furtive nature, whereas reindeer tend to display more placid and approachable behaviors (Ingold, 1974). This contrast in behavior raises intriguing questions about the genetic mechanisms underpinning the docility of reindeer, and whether this inherent docility played a role in their successful domestication. The genetic basis behind this docility, particularly in the context of domestication, remains largely unexplored and could provide important insights into the evolutionary pathways that led to the domestication of some species in the Cervidae family.

The aim of this study was sought to elucidate the genetic basis of different domestic reindeer populations by resequencing 32 reindeer genomes from wild and domestic populations across a broad geographic range, namely Canada, Norway, Siberia, and China. Our findings will contribute to a better understanding of the demographic history of reindeer and the genetic basis of domestication.

## Methods

### Sample collection and sequencing

A total of 32 reindeer blood or muscle samples were collected and sequenced, including 9 wild and 23 domestic reindeer. The wild reindeer were collected from two areas, including W_Quebec (four samples) from Quebec region of Canada and W_Norway (five samples) from Snøhetta mountain region of south-central Norway. The domestic reindeer were collected from three areas, including D_China (10 samples) from the Greater Xing’an Mountains area of China, D_Siberia (4 samples) from the Siberian Yamal-Nenets autonomous region, and D_Norway (9 samples) from Filefjell reindeer husbandry area in south-central Norway. Unfortunately, we were unable to include wild reindeer samples from Asia in our study, primarily due to the significant decline in their populations (Bakka, et al. 2022; Wang et al., 2019).

DNA extraction from fresh muscle or blood tissues was performed using Gentra Puregene Blood Kit (Qiagen). DNA quality and integrity were checked by A260/A280 and agarose gel electrophoresis. And then the extracted DNA was then fragmented into smaller fragments using sonication. Subsequently, the fragmented DNA underwent end repair using T4 DNA polymerase (Thermo Scientific) and Klenow fragment, followed by phosphorylation with T4 polynucleotide kinase (Thermo Scientific) to repair and modify the DNA ends. To add adenine bases to the 3ʹ ends of the DNA fragments, an A-tailing step was carried out using a polymerase and dATP. Adapter ligation was accomplished by hybridizing sequencing adapters containing specific primer sequences for downstream amplification and sequencing to the A-tailed DNA fragments. Covalent linking of the adapters to the DNA fragments was achieved using T4 DNA Ligase (Thermo Scientific). The resulting DNA library was then amplified via polymerase chain reaction using primers designed to hybridize with the adapter sequences, thereby enriching the DNA library and increasing the amount of DNA available for sequencing. Quality control of the constructed DNA library involved quantification using fluorometric assays and size analysis using agarose gel electrophoresis. Finally, the DNA library was loaded onto an Illumina sequencing instrument (Illumina HiSeq 2000) system, enabling high-throughput sequencing utilizing reversible terminator chemistry.

### Reads mapping and variant calling

We first downloaded the reindeer genome (GCA_022457185.1) from NCBI (Lin et al., 2019). Then Fastp (Chen et al., 2018) was used with default parameters to remove low-quality reads. The high-quality reads were mapped onto the reindeer reference genome using Burrows-Wheeler Aligner (BWA) (v0.7.17-r1188) (Li & Durbin, 2009), with the parameter “BWA mem -t 10 –R.” SAMtools (v1.9) (Li, 2011) was used to sort and merge files and convert mapping results into Binary Alignment Map (BAM) format. Local realignment, duplicate read marking were processed using the Picard (release 2.1.1, http://broadinstitute.github.io/picard/). The variants calling for each sample was performed using the Genome Analysis Toolkit (GATK) v4.1.1.0 (McKenna et al., 2010). In brief, the variants were called for each accession by GATK HaplotypeCaller. In addition, a joint genotyping step for comprehensive variations union was performed on Genome Variant Call Format file (gVCF) files. To remove the potential false positive single-nucleotide polymorphisms (SNPs), SNPs with Quality-by-Depth (QD) < 2.0 or FisherStrand (FS) > 60.0 or RMSMappingQuality (MQ) < 40.0 or MQRankSum < −12.5 or ReadPosRankSum < −8.0 were filtered. Moreover, SNPs were retained only if they could be genotyped in at least 90% individuals of the samples used for SNP calling.

### Population genetic analysis

To identify closely related individuals, the KING (v 2.2.4) (Manichaikul et al., 2010) was used to estimate kinship coefficients for all pairs of samples. Maximum likelihood (ML) tree was constructed with iqtree2 (Minh et al., 2020) with default parameters. FigTree v.1.4.3 (http://tree.bio.ed.ac.uk/software/figtree/) was used to display the ML tree. Principal component analysis (PCA) of whole-genome SNPs for all 28 individuals was performed by PLINK with the parameter “--pca.” Furthermore, population structure was assessed using the default setting in the ADMIXTURE (Alexander & Lange, 2011) v.1.3.0. The number of assumed genetic clusters *K* ranged from 2 to 5.

### Genome-wide patterns of reindeer population genetics

In order to describe the genetic diversity within groups, we used VCFtools v0.1.17 (Danecek et al., 2011) to calculate the nucleotide polymorphism (π) and intergroup fixed index (*F*_ST_) of 50-kb windows with 10 kb steps. In addition, we used PLINK to calculate runs of homozygosity (ROH) and coefficient of inbreeding (*F*). The parameter *r*^2^ for linkage disequilibrium (LD) was calculated for pairwise SNPs within each scaffold using PLINK with the parameters “--*r*^2^ --ld-window-kb 501 --ld-window 99999 --allow-extra-chr --ld-window-*r*^2^ 0.” The average *r*^2^ values were calculated for each length of distance, and the whole-genome LD was averaged across all scaffolds. The LD decay plot was depicted against the length of distance using the R script (http://www.r-project.org). And we use an in-house script to calculate heterozygosity.

### Genome-wide selective sweep scans

To identify the potential regions putatively under selection, nucleotide diversity (π), and population fixation statistics (*F*_ST_) were calculated using VCFtools (Danecek et al., 2011). π is the expected heterozygosity per site derived from the average number of sequence differences in a group of samples, to detect genomic regions under selection during domestication, we calculated the ln ratio of (π_domestic_/π_wild_), (π_D_Norway_/π_W_Norway_), (π_D_Siberia + D_China_/π_W_Quebec_), and *F*_ST_ of 50-kb windows with 10-kb steps. And *F*_ST_ used for estimating the degree of pairwise genomic differentiation between wild and domestic reindeer. Moreover, SweeD v.3.2.1 (Pavlidis et al., 2013) was used for detecting complete selective sweeps with default settings implements the composite likelihood ratio method, which identifies regions with significant deviations from the neutral site frequency spectrum (SFS). We employed the Monte Carlo method with 10,000 iterations to assess the statistical significance of the overlap among the triple datasets.

### Demographic history

The pairwise sequentially Markovian coalescence (PSMC) model (Li & Durbin, 2011) was used to infer the history of the ancestral population dynamics of reindeer. PSMC reconstructed the historical changes of the effective population size with time according to the distribution of the most recent common ancestor between two alleles of an individual. In order to understand whether all individuals in the group have a consistent history of reindeer, we used all wild reindeer individuals and domestic reindeer individuals, first using SAMtools mpileup to obtain the consensus sequence of each individual. Then set the PSMC parameter to “-u 8.42 × 10^-9^ -g 4 -Y 30 -X 1e7 -p” to reconstruct the group history. The average generation time is set to 4 years, and μ (unit: nt/generation) is set to 8.42 × 10^−9^ according to the values provided in the previous literature (Lin et al., 2019). PSMC modeling uses the bootstrap method, and each sample is run with 100 iterations to estimate the variance of the simulation results. We also used the multiple sequentially Markovian coalescent (MSMC2) to calculate the relative cross-population coalescent rate (rCCR) with default parameter. rCCR of 0.5 was consider as an indicator of population split.

Fastsimcoal2 speculates on population differentiation time and effective population size based on frequency spectrum (SFS). We then used Fastsimcoal2 (Excoffier et al., 2021) to simulate the recent population history of reindeer with the program 30 times for each model to ensure convergence and maximize the likelihood of fitting. Each likelihood is obtained after 100,000 simulations (−n100,000, −N100,000), −L is set to 40, which is 40 expectation/conditional maximization cycle.

### Identification of conserved noncoding elements

We downloaded 16 vertebrate genomes from NCBI and ENSEMBL 104, including reindeer (GCA_022457185.1), white tailed deer (GCA_002102435.1), roe deer (GCA_951849835.1), pronghorn (GCA_007570785.1), springbok (GCA_006408585.1), cow (GCA_002263795.3), goat (GCA_001704415.1), sperm whale (GCA_002837175.2), horse (GCA_002863925.1), human (GCA_000001405.29), platypus (GCA_004115215.2), chicken (GCA_016699485.1), common wall lizard (GCA_004329235.1), tropical clawed frog (GCA_000004195.4), lungfish (GCA_019279795.1), and coelacanth (GCA_000225785.1) (Chen et al., 2019, 2022; Low et al., 2020; Wang et al., 2021; Zhou et al., 2021). Using the reindeer genome as a reference, whole-genome alignments were generated for these 16 species. The methods in detail are the same as in previous studies (Frith & Ni, 2023; Huang et al., 2023; Wang et al., 2023; Wu et al., 2021, 2023); LASTZ (Nahar et al., 2021) was first used for alignments with maf format, and MULTIZ (waterman & bell, 1984) was used to integrate the alignments for different species. Then, those noncoding sequences remained in other 15 vertebrates with over 80% similarity, but lost in reindeer genome, may be the reindeer-specific missing conserved noncoding elements (CNEs). If at least 10 or more contiguous variant sites are present in reindeer sequences, but they are highly conserved in the other 15 species, and the conservation of these sequences is >80% in these 16 species, such sequences could be potentially divergent CNEs. The corresponding scripts can be found at https://github.com/wubaosheng/conserved-non-coding-elements-finding. We downloaded the human H3K27ac and H3K27me3 ChIP-seq data for visualization from http://epigenomegateway.wustl.edu/browser/ to further investigate whether these regulatory regions have chromatin accessibility.

### Identification of reindeer-specific amino acid substitutions

As we did above to identify reindeer-specific CNEs following our previous study (Wu et al., 2021, 2023), 16 vertebrate protein sets were used to identify reindeer-specific amino acid (AA) substitutions. We first constructed a protein set for the one-to-one homologous gene of these species using the reciprocal best hit (RBH) approach and scanned each AA site for each homologous gene. The AA site was considered a reindeer-specific substitution site if it had a consistent AA in the other 15 species, but different AAs in reindeer, and the similarity of the 10 sites upstream and downstream of that AA site was greater than 60%.

## Results and discussion

### Genome resequencing

**Figure 1.**
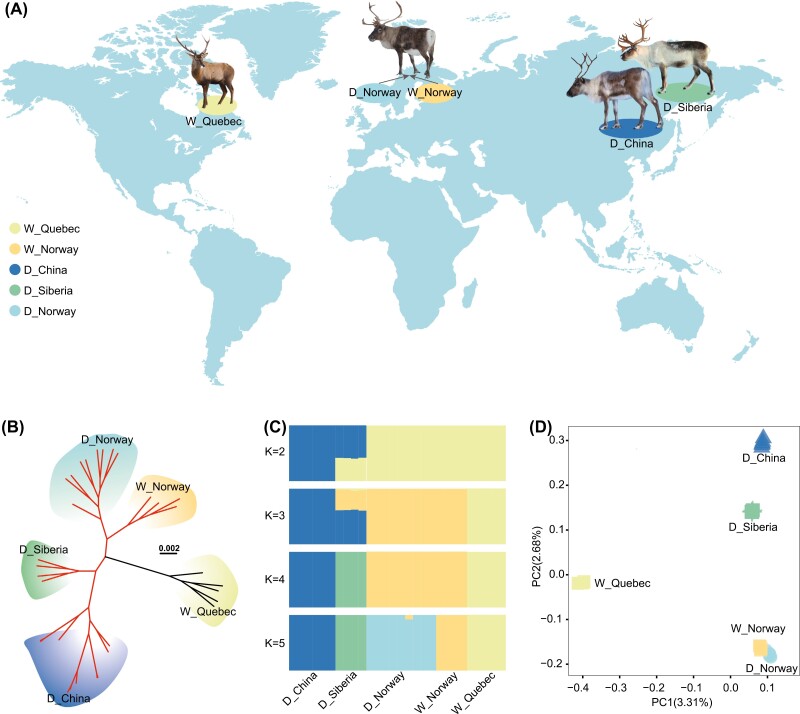
Phylogenetic and population genetic analyses of wild and domestic reindeer. (A) Geographic distribution of the sampling locations for two wild reindeer populations (W_Quebec and W_Norway) and three domestic reindeer populations (D_Siberia, D_China, and D_Norway). (B) Maximum likelihood phylogenetic tree constructed using whole-genome single-nucleotide polymorphism (SNP) data. Colors indicate different populations (W_Quebec, W_Norway, D_Siberia, D_China, D_Norway). Red branches indicate Eurasian populations and black branches indicate North American populations. (C) Model-based clustering by admixture with the number of ancestry kinships (*K*) set as 2, 3, 4, or 5. Vertical bars indicate the proportion of genetic ancestry from each ancestral population. (D) The principal component analysis of the first 2 components of 32 reindeer samples. The color and shape of dots indicate the different populations.

To investigate the genetic basis of reindeer domestication, we collected 32 samples from wild and domestic reindeer from 5 different geographic populations covering the main distribution areas of reindeer (Figure 1A), namely two wild populations from Norway (W_Norway) and northern Quebec (W_Quebec) in Canada, and 3 domestic populations from Norway (D_Norway), Siberia (D_Siberia), and China (D_China). After filtering the raw reads, we obtained 1.21 Tb high-quality clean reads for further analysis. The average depth was 10.59×, the average mapping rate of reads to our released reference genome was 98.54%, and the average genome coverage was 94.72% (Supplementary Table S1). We also detected 29.91 million high-quality SNPs in the sequenced reindeer genomes, 72.8% of them were in intergenic regions (Supplementary Table S2), which is similar to the SNP distribution in yak and cattle genomes (76.4% in intergenic regions in yak [Qiu et al., 2015] and 70.7% in intergenic regions in cattle [Choi et al., 2014]).

### Population structure and evolutionary history

A ML tree was reconstructed based on the high-quality SNPs found in the 32 wild and domestic reindeer genomes (Figure 1B). The results showed that the W_Quebec populations formed one clade (named the North American caribou), and all the other populations formed another clade (named the Eurasia reindeer group), which was further divided into two subgroups, Norwegian and Siberia reindeer groups (Figure 1B). The admixture analysis (*k* = 3) grouped the samples into three different clusters: North America, Asia, and western Europe (Figure 1C), which is in accordance with the ML tree structure. The relationships of these different populations were confirmed by PCA (Figure 1D); the first principal component separated the W_Quebec population from the other four populations and the second component separated the W_Norway, D_Norway, D_Siberia, and D_China populations. These results suggest that two or more independent domestication events occurred for reindeer in northern Europe and Siberia.

**Figure 2.**
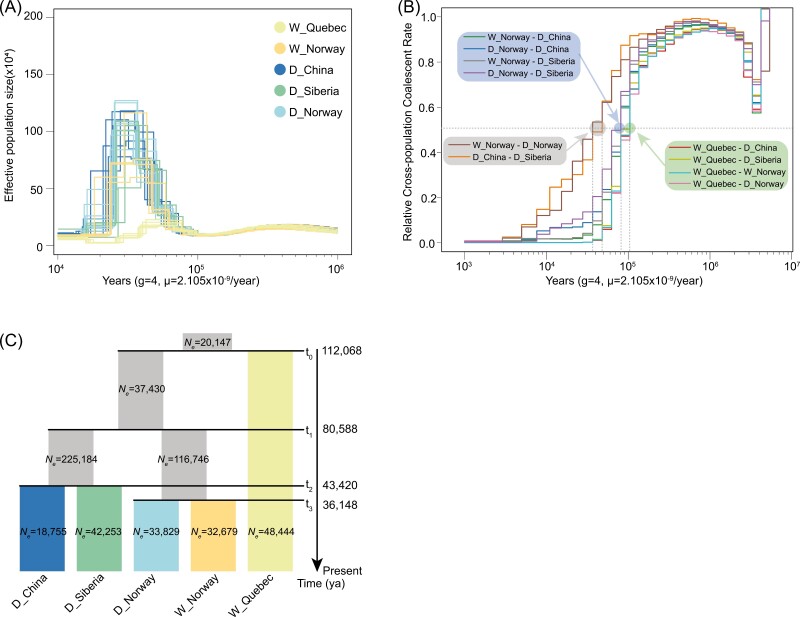
Divergent time and demographic history of reindeer. (A) Demographic history inferred by pairwise sequentially Markovian coalescence (PSMC). Colors represent different populations (W_Quebec, W_Norway, D_Siberia, D_China, and D_Norway). (B) Relative cross-coalescence rates for five population pairs estimated by MSMC2. Dotted lines indicate the split time for the population pair (relative cross-coalescence rates = 0.5). Colored lines indicate relative cross-coalescence rates between different population pairs. (C) Schematic representation of the demographic scenario modeled in fastsimcoal2. Estimated effective population sizes (*Ne*) and divergence times are indicated for different reindeer populations. Arrows indicate the migration for different reindeer groups.

We then performed a PSMC (Li & Durbin, 2011) to evaluate the effective population size (*Ne*) of the ancestral population for both wild and domestic reindeer in response to Quaternary climatic change (Figure 2A) based on a mutation rate of 2.105 × 10^−9^ per site per year and generation time of 4 years (Lin et al., 2019). We found that the ancestors of today’s reindeer populations had similar demographic patterns before 100,000 years ago. After this period, these ancestral groups, excluding the lineage that culminated in the present-day reindeer population of caribou, underwent a period of rapid expansion with effective population size peaking ~40,000 years ago. We also performed MSMC2 analysis (Schiffels & Wang, 2020) to estimate divergence times using the same mutation rate and generation time as we used for the PSMC analysis (Figure 2A and B). The divergence time of the ancestral state of W_Quebec and other populations was ~110,000 years ago. The divergence time of the ancestral state of W_Norway, D_Norway, D_China, and D_Siberia was roughly 80,000 years ago. The ancestral state of W_Norway and D_Norway diverged approximately 35,000 years ago, and that of D_China and D_Siberia diverged approximately 40,000 years ago (Figure 2B and C). The deep divergence times that we found in the reindeer populations may be linked to climatic and environmental changes during the LGP when the harsh conditions may have led to the formation of refugial populations, isolating groups of reindeer and contributing to genetic divergence (Klütsch et al., 2016; Polfus et al., 2017; Yannic et al., 2014). After the LGM, as the climate warmed and habitats expanded, the isolated populations may have come into contact again, leading to the genetic patterns that are found in today’s populations (Yannic et al., 2014). In addition, the end of the LGM coincided with significant shifts in ecosystems and animal populations (Klütsch et al., 2016), potentially influencing the domestication of reindeer. To further elaborate, the post-LGM period marked a transition to more stable and warmer climatic conditions, which facilitated the expansion of human settlements and the development of agriculture (Fabrice, 2019). This socioenvironmental shift likely provided opportunities for closer interactions between humans and reindeer, setting the stage for the eventual domestication of these animals. Understanding this connection helps to contextualize the genetic patterns we have been discovered in the context of significant historical and environmental changes.

The divergence time between the wild North American reindeer group (caribou) and the Eurasian reindeer group that we obtained deviates from divergence times obtained previously using mitochondrial data. For example, 184,000–430,000 years before present (BP) based on microsatellite and mitochondrial cytochrome b (Yannic et al., 2014), 44,600–135,800 years BP based on whole mitochondrial genomes (Klütsch et al., 2016), 68,600–173,600 years BP with microsatellite data and mitochondrial DNA control region haplotypes (Polfus et al., 2017), and 28,100-46,700 years BP with control region only (McDevitt et al., 2009). Because the unidirectional replication of the mitochondrial genome, which differs from the replication of the nuclear genome, involves intricate regulatory and repair mechanisms, the rate of evolutionary change determined based on nuclear and mitochondrial genomes is different (Allio et al., 2017). This disparity in evolutionary rates may have contributed to the differences in estimated divergence times of reindeer populations between our findings and those of other studies.

### Weak artificial selection pressure in domestic reindeer

**Figure 3.**
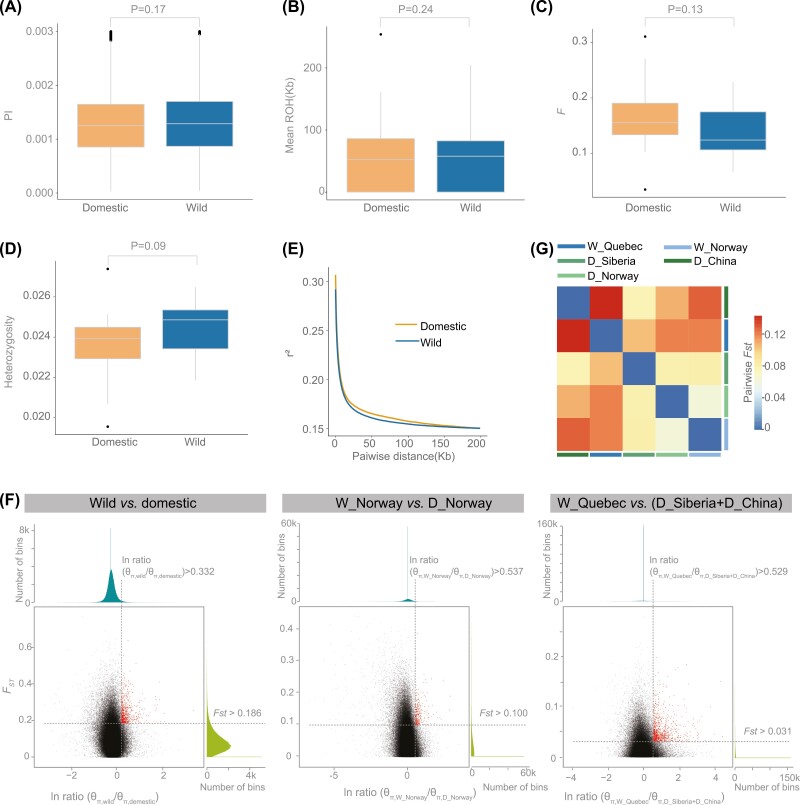
Estimates of genetic diversity summarized for wild and domestic reindeer. (A) Nucleotide diversity (π) of wild and domestic reindeer. (B) Mean length of runs of homozygosity (ROH). (C) Coefficient of inbreeding of wild and domestic reindeer. (D) Heterozygosity of wild and domestic reindeer. (E) Decay of linkage disequilibrium (LD) of wild and domestic reindeer populations measured by the average LD coefficient *r*^2^. (F) Distribution of ln ratio of (π_domestic_/π_wild_), (π_D_Norway_/π_W_Norway_), (π_D_Siberia + D_China_/π_W_Quebec_) and *F*_ST_ of 50-kb windows with 10 kb steps. Red dots indeicate windows fulfilling the selected regions requirement (corresponding to *Z* test *p* < 0.05, where *F*_ST_ > 0.186 and ln ratio > 0.332 for domestic vs. wild subgroups; *F*_ST_ > 0.100 and ln ratio > 0.573 for W_Norway vs. D_Norway subgroups; *F*_ST_ > 0.031 and ln ratio > 0.592 for W_Quebec vs. (D_Siberia + D_China) subgroups). (G) Correlation heatmap of pairwise *F*_ST_ between each two populations of the total five populations in this study based on R software.

To assess the effects of artificial selection on domestic populations, we estimated the nucleotide diversity (π), average ROH length, inbreeding coefficient (*F*), and heterozygosity for the wild and domestic populations in this study. Surprisingly, we found no significant divergence between wild and domestic reindeer using these parameters (Figure 3A–D and Supplementary Table S3), implying that the patterns of genomic diversity between wild and domestic reindeer are similar. We also estimated LD in wild and domestic reindeer based on the average LD coefficient (*r*^2^) for SNPs within 100-kb windows. We found that LD decreased to half its maximum value at 149 kb (*r*^2^ = 0.15) and 150 kb (*r*^2^ = 0.15) in domestic and wild reindeer, respectively (Figure 3E), indicating that LD had similar rates of decline in domestic and wild reindeer.

We then examined the distribution of π ln-ratios for wild vs. domestic populations to evaluate the distribution of candidate selective regions on the domestic reindeer genomes. Because the genetic structure of the five populations divided into three subgroups (North America, Asia, and Europe), we examined the distribution of wild vs. domestic π ln-ratios in three comparison groups, namely (D_Norway + D_China + D_Siberia) vs. (W_Quebec + W_Norway), D_Norway vs. W_Norway, and (D_Siberia + D_China) vs. W_Quebec (Figure 3F). Our results indicate that none of the large regions had significantly higher π ln-ratios, and the number of regions with significant higher π ln-ratios was almost the same. The density distribution of the π ln-ratios in the three comparison groups also showed that very few π ln-ratios had significantly higher values (Figure 3F). Furthermore, the Pearson correlation coefficient for pairs of π ln-ratios from the three wild vs. domestic comparisons showed that the correlation between two groups was very low (Supplementary Table S4), implying that the nucleotide diversity of each population was not significantly changed.

We also calculated pairwise *F*_ST_ values to determine population differentiation among different populations (Figure 3G). The *F*_ST_ values between wild and domestic populations were 0.062–0.145, indicating no significant difference between wild and domestic populations. The average *F*_ST_ (*F*_ST_ = 0.08) between wild and domestic populations was also smaller than the average *F*_ST_ reported for many other wild and domestic species, including taurine vs. zebu cattle, diverged taurine cattle breeds (*F*_ST_ = 0.215) (Porto-Neto et al., 2014), dogs vs. wolves (*F*_ST_ = 0.223) (Axelsson et al., 2013), Asiatic mouflon vs. landraces of sheep (*F*_ST_ = 0.125), and Asiatic mouflon vs. improved breeds (*F*_ST_ = 0.132) (Li et al., 2020). These results reflect weak artificial selection pressure in domestic reindeer populations.

We further examined whether specific genomic regions in the domestic population had signals of artificial selection, and found a small number of regions with greater differentiation and lower polymorphism in the domestic population compared with the wild population. In the three wild vs. domestic comparison groups, we identified 729, 333, and 980 regions where artificial selection signals seemed to be present (Figure 3F, Supplementary Tables S5–S7), but only nine of these regions overlapped; five covered the protein kinase gene *SRPK1*, one covered the adducin gene *ADD2*, and the other three were on short scaffolds with no annotated genes. To investigate whether these overlapping regions were significant or randomly generated, we analyzed the significance of the results generated by these three methods and found that the overlapping regions were randomly generated (*p-*value > 0.1). We also found that *SRPK1* and *ADD2* were not significantly associated with relevant biological functions (Mytilinaios et al., 2012; Porro et al., 2010).

### Genetic basis for the docile nature of reindeer

**Figure 4.**
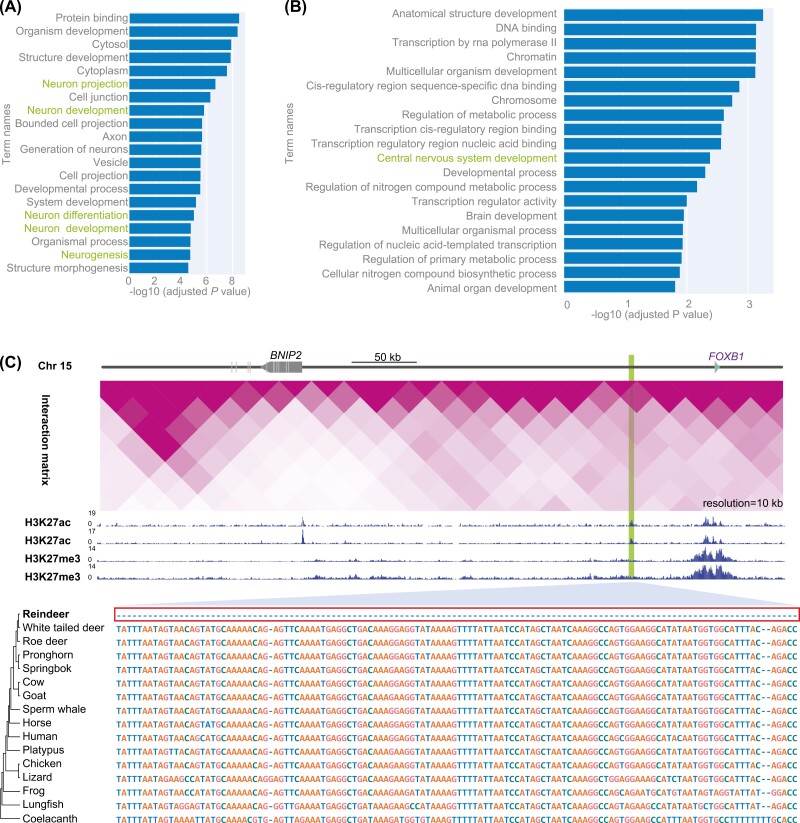
Genetic variation in the reindeer genome underlying the ease of domestication of reindeer. (A) Gene ontology (GO) enrichment for 344 genes with reindeer-specific amino acid (AA) substitutions. The top 20 significantly enriched GO terms (adjusted *p*-value < 0.01) are shown. (B) GO enrichment of the neighboring genes of the loss and divergent conserved noncoding elements (CNEs). The top 20 significantly enriched GO terms (adjusted *p*-value < 0.01) are shown. (C) Interaction of *FOXB1* with surrounding CNEs in the human genome and cross-species sequence alignments. Hi-C data show that *FOXB1* interacted strongly with the upstream CNE. ChIP-seq data for H3k27ac and H3k27me3 show that this CNE may be an important enhancer. Multiple sequence alignment of 15 vertebrate sequences shows that this CNE is highly conserved across species.

Our findings suggest that reindeer domestication involved limited anthropogenic intervention and that the process was influenced significantly by the inherent traits of reindeer. Next, we traced genetic alterations that occurred in reindeer and 11 other mammals, especially Cervidae species, and identified a total of 344 genes with reindeer-specific AA substitutions in at least one conserved region (Supplementary Table S8). Interestingly, the gene ontology (GO) enrichment showed that nervous system-related GO terms (GO:0043005, GO:0048699, GO:0030182, GO:0031175, GO:0022008, and GO:0007399) were significantly enriched for those genes (Figure 4A and Supplementary Table S9).

In addition, compared with the genome of other Cervidae species, 77 CNEs were absent and 159 CNEs were divergent in the reindeer genome (Supplementary Table S10), implying that specific changes in these CNEs may have occurred in the reindeer genome. The GO enrichment analysis of the genes that surrounded the divergent CNEs indicated that they were related to nervous system development (Figure 4B and Supplementary Table S11) (e.g., *FOXB1*, *HOXA3*, *PBX1*, *NAV3*, and *NDEL1*). For example, one of the most highly conserved CNE (~140 bp, 96.4% similarity in mammal genomes) was located about 58 kb upstream of *FOXB1* and the Hi-C data showed that there was strong interaction between the upstream CNE and *FOXB1* (Figure 4C). The ChIP-seq data for histones H3K27ac and H3K27me3 in human brain showed that this CNE might be an enhancer for *FOXB1* (Figure 4C). *FOXB1* is an important regulator of oligodendrocyte differentiation and maturation in the central nervous system, and its loss could usually results in mental retardation (Zhang et al., 2017). Thus, the loss of the *FOXB1*-related enhancer and other CNEs in the reindeer genome may cause the reindeer nervous system to be relatively unresponsive, thus facilitating domestication.

Combining the results of previous studies (Lin et al., 2019), we further compared the PI and *F*_ST_ values of 344 gene regions with reindeer-specific AA substitution, 130 positively selected genes, and 159 divergent CNEs, but found no significant difference between the wild and domestic populations (*p-*value = 0.4487). This finding further supports the proposal that characteristics of the reindeer species itself are key factors in domestication.

## Concluding remarks

The whole-genome resequencing analysis of 32 reindeer samples from domestic and wild populations has provided new insights into the genetic basis of reindeer domestication. Excluding the domesticated reindeer that were introduced into North America during the 18th and 19th centuries (Jepsen et al., 2002; Lantis, 1950; Mager, 2012), our results show that at least two independent domestication events contributed to the current distribution of domestic reindeer populations (Figure 1B–D). The divergence time between North American and Eurasian reindeer populations, which was calculated using genomic SNPs, was different from the divergence time estimated using the mitochondrial genome (Figure 2A and B) (Yannic et al., 2014). This discrepancy may be attributed to the different evolutionary rate exhibited by the mitochondrial and nuclear genome (Allio et al., 2017).

Surprisingly, the genetic diversity and LD found little difference between wild and domestic reindeer populations (Figure 3A–G). Population differentiation as measured by *F*_ST_ was also lower than that of other domesticated species (Figure 3F). Together, these results imply only weak artificial selection pressure during reindeer domestications. However, by comparing the reindeer genomes with other Cervidae genome, we identified genes and noncoding elements with reindeer-specific changes that were enriched in GO terms related to nervous system. This finding suggests that inherent physiology and behavior traits of reindeer, rather than artificial selection, facilitated their domestication. The proposition that changes in the nervous system may have played a role in the domestication of wild animals is supported in the context of cats (*Felis catus*) and dogs (*Canis lupus familiaris*). (Montague et al., 2014; Wilkins et al., 2014). Interestingly, the GO enrichment analysis indicated that genes and putative enhancers that exhibited reindeer-specific changes were involved in neuronal maturation and the central nervous system. One of the genes was *FOXB1*, an important regulator of oligodendrogenesis (Zhang et al., 2017), and loss or divergence of the putative enhancer of *FOXB1* may have contributed to a less responsive nervous system in reindeer. Combined with previous findings (Lin et al., 2019), our finding supports the proposals that the docile behavior of reindeers arose from inherent neurological traits that pre-adapted them for human management and domestication.

Overall, our genome-wide study sheds new light on the genetic mechanisms underlying reindeer domestication. Unlike the strong artificial selection seen in other domesticated species (Axelsson et al., 2013; Montague et al., 2014; Wilkins et al., 2014), reindeer seem to have been domesticated primarily due to their own unique neurological predispositions. Further functional studies are needed to the validate putative selective targets and elucidate their specific roles in reindeer behavior and domestication.

## Supplementary Material

qrae006_suppl_Supplementary_Tables_S1-S11

## Data Availability

The raw data of resequencing of reindeer genomes have been deposited in the NCBI under accession number PRJNA974187.
